# 

**Published:** 1907-09

**Authors:** 


### BOOKS RECEIVED.

Surgery: Its Principles and Practice. In five volumes. By 66 eminent surgeons. Edited by W. W. Keen, M.D., LL.D., Hon. F.R.C.S., Eng. and Edin., Professor of the Principles of Surgery and of Clinical Surgery, Jefferson Medical College, Phila. Volume II. Octavo of 920 pages, with 572 text-illustrations and 9 colored plates. Philadelphia and London: W. B. Saunders Company. 1907. (Cloth, $7.00; half morocco, $8.00 net prices.)

---

Transactions of the Southern Surgical and Gynecological Association. Vol. XIX. Nineteenth annual session held at Baltimore, December 11-13, 1906. W. D. Haggard, M.D., Secretary.

---

Physicians' Manual of the Pharmacopeia and the National Formulary. An Epitome by C. S. N. Hallenberg, Ph.G., M.D., Professor of Pharmacy in the School of Pharmacy, University of Illinois. Chicago: American Medical Association. (Price, 50 cents.)

---

Manual of the Diseases of the Eye. By Charles H. May, M.D., Instructor in Ophthalmology, College of Physicians and Surgeons, New York. Fifth edition. 12 mo, pp. 391. Illustrated. New York: William Wood & Co. (Price, $2.00.)

---

Cook County Hospital Reports. 1906. Compiled and edited under the direction of the publication committee of the Cook County Hospital attending staff.

---

A Treatise on Fractures and Dislocations. By Lewis A. Stimson, B.A., M.D., Professor of Surgery in Cornell University Medical College, New York. New (5th) edition, thoroughly revised. Octavo, 847 pages, with 352 engravings and 52 plates. Lea Brothers & Co., Philadelphia and New York, 1907. (Cloth, $5.00, leather, $6.00, half morocco, $6.50, net prices.)

